# Dietary fat intake and quality in long-term care residents in two cohorts assessed 10 years apart

**DOI:** 10.1186/s40795-022-00524-9

**Published:** 2022-04-12

**Authors:** K. Jyväkorpi Satu, H. Suominen Merja, E. Strandberg Timo, Karoliina Salminen, T. Niskanen Riikka, Hanna-Maria Roitto, K. T. Saarela Riitta, H. Pitkälä Kaisu

**Affiliations:** 1 Department of General Practice and Primary Health Care, and Helsinki University Central Hospital, Unit of Primary Health Care, University of Helsinki, Tukholmankatu 8, 00014 Helsingin yliopisto Helsinki, Finland; 2 Center for Life Course Health Research, University of Oulu, Oulu, Finland; 3 City of Helsinki, Department of Social Services and Health Care, Geriatric Clinic, Helsinki Hospital, Helsinki, Finland; 4 City of Helsinki, Department of Social Services and Health Care, Oral Health Care, Helsinki, Finland

**Keywords:** Fat quality, Fat composition, Saturated fatty acids, Monounsaturated fatty acids, Polyunsaturated fatty acids, Long-term care

## Abstract

**Purpose:**

To describe and compare detailed dietary fat intake, fat quality and associative factors between two measuring points 10 years apart of residents living in long-term care facilities, and to reflect how fat composition and fat quality corresponds to current nutrition recommendations.

**Methods:**

In 2007 long-term care residents (*n* = 374) of 25 assisted-living facilities and nursing homes and in 2017–18 long-term care residents (*n* = 486) of 17 respective facilities in Helsinki metropolitan area were recruited for this study. Information on the residents’ heights, demographic information and use of calcium and vitamin D supplementation were retrieved from medical records. Residents’ clinical assessment included Clinical Dementia Rating (CDR), the Mini Nutritional Assessment (MNA) and questionnaire related to nutrition care. Participants’ energy and fat intake were determined from 1--2-day food diaries kept by the ward nurses, and fat quality indicators calculated.

**Results:**

Age, gender distribution, MNA score or body mass index did not differ between the two cohorts. Residents’ cognitive status, subjective health and mobility were poorer in 2017 compared to 2007. Total fat and saturated fatty acid (SFA) intakes were higher and fat quality indicators lower in the 2017 cohort residents than in the 2007 cohort residents. Sugar intake, male gender, eating independently, eating larger amounts and not having dry mouth predicted higher SFA intake in the 2017 cohort.

**Conclusions:**

The fat quality in long-term care residents in our study worsened in spite of official recommendations between the two measurement points.

## Background

Fat is an important macronutrient in the human diet. It is a major source of energy and it facilitates the absorption of fat-soluble vitamins, carotenoids and other phytochemicals [1]. It is also a source for essential fatty acids (EFA) present particularly in vegetable oils, nuts, seeds and fish, which are necessary for development, normal function of nervous and cardiovascular systems and general health [2]. EFAs cannot be synthesized by humans and must be provided by the diet [3]. Fat also contributes to the acceptability and texture of foods, and improves taste by enhancing flavor and aroma.

Institutionalized older people often have cognitive and physical impairments along with many chronic illnesses [4]. Malnutrition is thus commonly encountered in long-term care residents and it weakens quality of life and increases morbidity and mortality [5–7]. Frailty, poor cognitive status, polypharmacy, gastro-intestinal symptoms, poor dentition, and swallowing difficulties often lead to inadequate energy and nutrient intakes, weight loss and malnutrition in long-term care residents [8–11]. It is a common practice in long-term care facilities to use more fat to prepare foods in order to increase acceptability and energy content of served foods [12]. Thus, fat has an important role in the diets of long-term care residents.

Recently in Finland, a special nutrition recommendation for older people was published, emphasizing along with sufficient protein intake, diet quality and other issues, the importance of fat quality also in the frailest older adults in long-term care [13]. In the recommendation -- similarly to many other national authorities’ and expert groups’ recommendations -- good fat quality is defined as keeping saturated fatty acid (SFA) intake lower than 10% of energy (E%) and replacing SFAs with monounsaturated fatty acids (MUFA) and polyunsaturated fatty acids (PUFA) [14–18]. Good fat quality has been previously shown to have an important role in cardiovascular and cognitive health, and may thus be important also for the health of the frailest older adults [14, 19, 20].

The purpose of this study was to describe and compare dietary fat intake, its detailed composition and quality and associative factors in two cohorts of residents living in long-term care facilities and in the same geographical area, measured 10 years apart, and to investigate how the fat composition and quality corresponds to the current nutrition recommendations.

## Methods

In 2007, a sample of long-term care residents from 25 assisted-living facilities and nursing homes in Helsinki metropolitan area were recruited for the original nutrition study. In 2017/18 we conducted a follow-up study and recruited volunteer residents from a sample of 17 assisted-living facilities and nursing homes in Helsinki. The long-term care facilities were randomly selected within voluntary institutions. Six of the original facilities were included in the 2017/18 sample. In all the institutions both in those included in 2007 and in 2017/18, registered nurses were in charge of the wards and constant 24/7 assistance was available.

The inclusion criteria for this present study were: age ≥ 65 years, living permanently in long-term care facility, sufficient information available on demographic factors and nutritional care, a filled 1--2-day food diary. Trained nurses collected the data both 2007 and 2017/18. The participants’ weights were measured. Their heights were obtained from the medical records, and body mass index (BMI) was calculated as weight divided by height squared (kg/m^2^). Information on the residents’ demographic information and use of calcium and vitamin D supplementation were retrieved from medical records. The cognitive status of the residents in both cohorts (2007, 2017/18) was measured using Clinical Dementia Rating (CDR) [21]. Mobility was assessed with one item in MNA questionnaire and catergorized as: bed or chair bound, able to get out of bed/chair, but does not go out or goes out. Similarly, self-rated health was also assessed by one item in MNA and categorized as: considers oneself healthy or quite healthy, considers oneself sick or very sick, not able to answer. Nutritional status was assessed, using the Mini Nutritional Assessment (MNA) long version [22]. In addition, several questions associated with nutritional care were asked. These included: 1) amounts of eaten foods (very little or little, normal or a lot); 2) whether resident eats snack (yes, no); 3) texture of food (liquid or puree, soft, normal); 4) use of oral nutritional supplements (yes, no); 5) frequency of weight monitoring (2–6 times a year, > 6 times a year); 6) chewing problems (yes, no); 7) dry mouth (yes, no); 8) pain in the mouth (yes, no); 9) dysphagia (yes, no); and 10) need of help with eating (yes, no).

Participants’ energy and detailed fat intakes were determined from 1--2-day food diaries kept by the ward nurses. Prior to the data collection, the nurses participated in comprehensive training sessions on how to fill food diaries for the residents organized by the study’s investigators (MS) in 2007 and (MS, SKJ) in 2017. In 2007, the food diaries were analyzed using Nutrica dietary software (version 3.11, Kela, Turku, Finland), and in 2017, using Aivo Diet dietary software (version 2.2.0.0, Aivo Oy, Turku, Finland) both containing the Fineli Food Composition database including foods and recipes for the typical Finnish mixed dishes that are customarily served in long-term care. The instruction was to record all the foods and beverages consumed by the resident. The nurses estimated portion sizes, using household measures. For prepacked products, the exact brand and product name were required. Dietary data including energy, total fat intake and fat composition of diet (including SFA, MUFA, and PUFA) were analyzed from food diaries. Using these data, we calculated fat quality indicators (MUFA:SFA- and PUFA:SFA- ratios), and percentage of energy from SFAs, MUFAs and PUFAs in the diets of the residents. In 2017, we also have data of more detailed fat composition, such as amounts of n-3 fatty acids, n-6 fatty acids, their ratios, or amounts of trans fatty acids. Due to limitations of the earlier dietary analyzing tool, these data were not available in 2007.

### Statistical analysis

The data from the two cohorts were combined and descriptive statistics presented. The differences between the baseline characteristics, energy and detailed fat intakes and fat quality of groups were analyzed between the two cohorts (2007, 2017), using the Chi^2^ -test or Fisher’s exact test for categorical variables and t-test or Mann Whitney U-test for continuous variables. SFA intake from 2017/18 cohort was divided into quartiles and nutrition related factors classified to those quartiles accordingly. The SFA intake quartiles of 2017/18 cohort were as follows; Q_1_ < 23.5263 g; Q_2_ = 23.5263 g--29.7475 g; Q_3_ = 29.7476 g − 37.7125 g; Q_4_ > 37.7125 g. Differences between nutritional factors classified into SFA quartiles were analyzed using Cochran-Armitage test for trend. Analysis of covariance (ANCOVA) test was used to investigate independent associations with SFA intake. Univariate general linear model was used to explore these associations. We explored associations of 2017 and of 2007 cohort separately. For the 2017 cohort: model 1 includes intercept, age, sex and sugar intake, and model 2 additionally includes need of help with eating (yes vs. no) and excludes age as a covariate. For the 2007 cohort: model 1 included the same characteristics that in 2017 cohort, in the model 2 additionally need of help with eating, and in the model 3 additionally total MNA score excluding need of help with eating and sex as covariates.

#### Selection of covariates

Age was selected as a covariate since older age is associated with more risk of malnutrition. Gender was selected as covariate, because females and males might differ in dietary preferences even in long- term care. Higher degree of dependency is a known risk factor for malnutrition. Thus needing help with eating was selected as a covariate [23] and total MNA score as a covariate, since it is a validated instrument to identify malnutrition [22]. Dietary sugar intake was selected as a covariate for the model because along with fat, increment of sugar is common in long- term care to increase acceptability and energy content of the diet and thereby prevent weight loss [24]. Accordingly, sugar in various forms sugary drinks and snacks; adding sugar to coffee, tea, porridge, and gruel, etc.) is frequently served. The statistical tests were performed using IBM SPSS statistical program (version 26, Chicago, US).

### Ethics

The ethics committee approvals were obtained for both 2007 and 2017/18 by the ethics committee of the Department of Medicine at Helsinki University Hospital and City of Helsinki (ethical approval number: HUS/2042/2016). Informed written consent was asked from all participants or in cases of moderate to severe dementia (MMSE < 20 points), from their closest proxies.

## Results

In total, 860 volunteer residents, which included 374 residents from 2007 cohort and 486 residents from 2017/18 cohort, were included in the study. Gender distribution, BMI nor MNA scores did not differ between the two cohorts (Table 1). Participants’ cognitive state measured using CDR, mobility and subjective health were poorer in the 2017/18 cohort compared to the 2007 cohort. Use of calcium supplements was more frequent in 2007 (47.2%) compared to 2017/18 (35.1%), *p* <  0.001, whereas vitamin D supplementation was more frequent in 2017/18 (82.5%) compared to 2007 (54.7%), *p* <  0.001.

**Table 1:** Baseline characteristics of two long-term care cohorts

Characteristics	Cohort of 2007;*n* = 374	Cohort of 2017/2018;*n* = 486	CI 95%	*p*-value^1^
Females, %	82.4	79.4		0.30
Age, years (SD)	83.3 (7.4)	82.4 (7.6)	−0.507, 1.505	0.33
MNA, total score (SD) Nutritional status (MNA), %	20.2 (3.5)	20.3 (3.4)	−0.588, 0.373	0.15
Good nutritional status, > 23	17.4	16.2		0.82
Risk of malnutrition 23.5–17	68.2	68.0		
Malnourished < 17	14.4	15.8		
BMI, kg/m^2^ (SD)	25.3 (4.8)	26.0 (5.0)	−1.339, 0.025	0.74
CDR, total score (SD) classification. %	1.8 (1.0)	2.6 (0.7)	−0.838, −0.610	< 0.001
0.5–1 Mild dementia	25.6	8.2		< 0.001
2 Moderate	39.5	26.5		
3 Severe	35.0	65.3		
Diabetes mellitus, %	16.2	16.6		0.45
Coronary heart disease, %	26.4	17.4		0.001
Coronary thrombosis, %	11.6	3.2		< 0.001
Stroke or TIA	24.1	22.9		0.38
Dementia	78.6	80.0		0.33
Subjective health, %				
considers oneself healthy or quite healthy	74.7	48.4		< 0.001
considers oneself sick or very sick	25.4	12.3		
Not able to answer	0	39.3		
Mobility, %				
bed or chair bound	13.6	47.1		< 0.001
able to get out of bed/chair but does not go out	45.5	26.4		
goes out	40.9	26.6		
Use of calcium supplementation, %	47.2	35.1		< 0.001
Use of vitamin D supplementation, %	54.7	82.5		< 0.001

*SD* Standard deviation, *CI* Confidence Interval, *MNA* Mini Nutritional Assessment, *BMI* Body mass index, *kg* kilogram, m meter; *CDR* Clinical Dementia Rating

^1^Statistical significance for *p*-value was set to < 0.05

Energy intake in females was lower in 2017/18 (1584 kcal) compared to 2007 (1653 kcal), whereas no differences were observed in energy intake in male residents between the two cohorts (Table 2). Total fat and SFA intakes were significantly higher in males and females in the 2017/18 cohort than in 2007, respectively, whereas the total amounts of MUFAs or PUFAs did not differ between the two cohorts in female or male residents.

**Table 2:** Energy, detailed fat intake and fat quality indicators between long-term cohorts of long term care residents in 2007 and 2017

Energy and fat intakes, fat quality and vitamins D and E intakes, mean (SD)	Cohort of 2007 *n* = 374	Cohort of 2017/2018 *n* = 486	CI 95%	*p*-value^1^	Nutrition Recommendation^a^
Energy, kcal (SD)	1691 (443)	1630 (397)	4.29, 118.71	0.04	
females	1653 (409)	1584 (393)	9.16, 129,13	0.02	
males	1870 (545)	1809 (363)	−90.89, 212.08	0.43	
Total fat, g (SD)	59 (21)	64 (20)	−7.80, −2.34	< 0.001	
females	57 (20)	61 (20)	−7.14, −1.19	0.006	
males	65 (22)	73 (18)	−13.75, −1.33	0.018	
SFA, g (SD)	24 (10)	31 (11)	−7.98, −5.20	< 0.001	
females	24 (10)	30 (10)	−7.40, −4.37	< 0.001	
males	26 (10)	35 (11)	−12.33, −5.79	< 0.001	
TRANS FA, g (SD)		1.3 (0.4)			
females	N/A	1.2 (0.4)			
males		1.4 (0.4)			
MUFA, g (SD)	18 (7)	19 (6)	−1.54, 0.19	0.13	
females	18 (7)	18 (6)	−1.50, 0.40	0.26	
males	21 (7)	21 (5)	−2.85, 1.27	0.45	
PUFA, g (SD)	7 (3)	7 (3)	−0.21, 0.62	0.33	
females	7 (3)	7 (2)	−0.28, 0.59	0.48	
males	9 (4)	8 (2)	− 045, 1.75	0.25	
N-3		3 (1)			
N-6	N/A	5 (2)			
N-6:N-3		1.7 (1)			
PUFA/SFA (SD)	0.32 (0.17))	0.24 (0.14)	0.05, 0.10	< 0.001	
females	0.31 (0.17)	0.24 (0.09)	0.05, 0.10	< 0.001	
males	0.34 (0.17)	0.26 (0.26	0.12, 0.16	0.022	
MUFA/SFA (SD)	0.78 (0.23)	0.63 (0.16)	0.12, 0.18	< 0.001	
females	0.78 (0.24)	0.63 (0.14)	0.12, 0.17	< 0.001	
males	0.81 (0.21)	0.64 (0.21)	0.11, 0.24	< 0.001	
FAT E%	31 E%	35 E%	−0.05, −0.03	< 0.001	25–40 E%
SFA E%	13 E%	17 E%	−0.05, − 0.04	< 0.001	< 10 E%
MUFA E%	9.7 E%	10.4 E%	−0.07, − 0.04	< 0.001	10–20 E%
PUFA E%	3.8 E%	4.3 E%	−0.01, --0.04	< 0.001	5–10 E%

*SD* Standard deviation, *CI* Confidence Interval, *SFA* Saturated fatty acids, *MUFA* Monounsaturated fatty acids, *PUFA* Polyunsaturated fatty acids, E% Percentage of total energy

^1^Statistical significance for *p*-value was set to < 0.05

^a^Nordic Nutrition Recommendation 2014

Fat quality indicators PUFA:SFA- ratio and MUFA:SFA-ratio differed significantly between the two cohorts. The females and males in the 2017/18 cohort had lower PUFA:SFA- ratios than observed in the 2007 cohort. Similarly, MUFA:SFA- ratios in both females and males were lower in the participants in the 2017/18 cohort compared to the 2007 cohort .

Mean PUFA n-3 intake was 3 g and n-6 intake 5 g in 2017/18 in participating residents, and ratio between n-3:n-6 was 0.6. We did not have this data from the 2007 cohort. Percentage of energy from total fat (Fat E%) and SFA were significantly higher in in the 2017/18 cohort than 2007 cohort, 35 E% and 31 E%, and 17 E% and 13 E%, respectively.

**Fig. 1:**
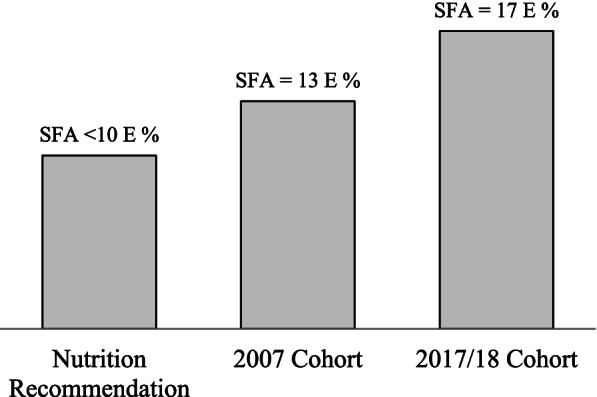
Saturated fatty acid (SFA) intake recommendation, mean SFA intake in 2007 and 2017/18 cohorts showed as percentage of energy (E%). E% = percentage of energy

The SFA intake quartiles were not associated with texture of the food, eating snacks, using oral nutritional supplements, more frequent weight monitoring, chewing problems, dysphagia, whereas they were linearly associated with the amount of eaten foods, and inversely associated with having dry mouth (Table 3). Those who had dry mouth consumed less energy than those who did not have dry mouth (1483 kcal vs. 1657 kcal). In addition, eating independently was linearly associated with higher SFA intake. Figure 1 illustrates how the SFA intakes are in line with nutrition recommendation.

**Table 3:** Percentage of nutrition related issues according to SFA quartiles in the long-term care cohort of 2017

SFA Quartiles	SFA Q_1_ *n* = 119	SFA Q_2_ *n* = 122	SFA Q_3_ *n* = 121	SFA Q_4_ *n* = 119	*p*- value^1^
Nutritional problems					
**Amount of eaten foods**, %					*p* < 0.001
very little or little	30	21	17	13	
normal	64	75	75	77	
a lot or very much	6	4	8	11	
**Eats snacks,** %					*p* = 0.76
yes	81	84	81	84	
**Texture of food,** %					*p* = 0.96
liquid, puree or soft	36	34	34	36	
normal	65	67	67	64	
**Uses oral nutritional supplements,**%					*p* = 0.22
yes	22	16	20	14	
**Weight monitoring,**%					*p* = 0.63
twice to six times a year	20	19	29	21	
> 6 times a year	79	81	71	79	
**Chewing problems,** %					*p* = 0.28
yes	29	30	24	24	
**Dry mouth,** %					*p* = 0.018
yes	18	17	12	8	
**Pain in the mouth,** %					*p* = 0.43
yes	10	6	5	8	
**Dysphagia,** %					*p* = 0.90
yes	19	14	16	18	
**Needs help eating, %**					*p* < 0.001
**yes**	83	75	71	63	

*SFA* Saturated fatty acids, *Q* Quartiles

^1^Statistical significance for *p*-value was set to < 0.05

In the ANCOVA model 1, dietary sugar intake and male gender were associated with higher SFA intake in the 2017 cohort, whereas age was not associated with SFA intake (adjusted *R*^2^ = 0.236). In the model 2, dietary sugar intake, eating independently and male gender were associated with higher SFA intake, adjusted (*R*^2^ = 0.244). In the 2007 cohort, in model 1 higher SFA intake was associated with sugar intake and age, whereas gender was not associated with SFA intake, adjusted *R*^2^ = 0.061 (Table 4). In the model 2 SFA intake was associated with sugar intake, age, whereas gender or eating independently were not associated with SFA intake (adjusted *R*^2^ = 0.065). In the model 3 SFA intake was associated with sugar intake, age and total MNA score (adjusted *R*^2^ = 0.068).

**Table 4:** Univariate general linear model of associative factors of saturated fat intake

2017 Cohort	B	95% confidence interval		***P***-value
		Lower Bound	Upper Bound	
**Model 1**				
Intercept	16.68	6.64	26.73	0.001
Age	0.26	−0.08	0.14	0.64
Sex (females vs. males)	−4.87	−6.94	−2.81	< 0.001
Sugar intake	0.13	0.11	0.15	< 0.001
Adjusted R^2^	**0.236**			
**Model 2**				
Intercept	16.86	6.08	27.63	0.002
Sex (females vs. males)	−4.86	−7.11	−2.61	< 0.001
Sugar intake	0.13	0.11	0.16	< 0.001
Need of help with eating (no vs. yes)	−3,17	−5.23	1.11	0.003
Adjusted R^2^	**0.242**			
**Model 3**				
Intercept	12.35	−0.53	25.23	0.060
Sex (females vs. males)	−4.77	−7.07	−2.47	< 0.001
Sugar intake	0.13	0.10	0.16	< 0.001
Total, MNA score	0.20	−0.08	0.48	0.164
Adjusted R^2^	**0.229**			
		**95% confidence interval**		
**2007 Cohort**	**B**	**Lower Bound**	**Upper Bound**	***P*-value**
**Model 1**				
Intercept	33.88	22.45	45.29	< 0.001
Age	−0.15	−0.29	−0.02	0.029
Sex (females vs. males)	−1.69	−4.34	0.97	0.21
Sugar intake	0.063	0.03	0.09	< 0.001
Adjusted R^2^	**0.052**			
**Model 2**				
Intercept	31.05	19.61	42.49	< 0.001
Age	−0.14	−0.28	−0.09	0.036
Sex (females vs. males)	−1.63	−4.25	0.99	0.22
Sugar intake	0.07	0.04	0.10	< 0.001
Need of help with eating (yes vs. no)	3.01	1.02	5.00	.003
Adjusted R^2^	**0.073**			
**Model 3**				
Intercept	25.41	11.82	39.00	< 0.001
Age	−0.14	−0.28	−0.01	0.038
Total MNA score	0.31	0.02	0.59	0.035
Sugar intake	0.07	0.03	0.10	< 0.001
Adjusted R^2^	**0.060**			

## Discussion

We observed that dietary fat quality in the long-term care facilities was poor in 2007 but even worse in 2017/18, in spite of the official nutrition recommendations. This worsening was due to significant increase of SFA intake and consequently dietary fat quality indicators worsened in older long-term care residents. Higher SFA intake of the 2017 cohort was predicted by sugar intake, male gender, eating independently, eating higher amounts of foods, and not having dry mouth.

Very few studies have described fat composition profile of long-term care residents. In a study by Rodríguez-Rejón et al. [25] fat composition of Spanish long-term care facilities was reported, but quality indicators were not calculated. In their study, SFA intake was about half of that in 2017/18 cohort in our study. In a Canadian study, SFA intake evaluated from long-term care facilities’ menus was about the same as in the 2007 cohort, but MUFA and PUFA intakes were considerably higher than in either of the cohorts in our study [26].

Recommendations on fat quality are uniform across various national authorities and expert groups [14–18]. Generally, all authorities encourage reducing SFA intake to less than 10 E% or even reducing it even more and replacing it with MUFAs and PUFAs [15]. In our study, fat quality in the long-term care residents was already poor in 2007, but even worse in 2017: SFA intake of the residents was on average 17 E% compared to the 13 E% in 2007. Moreover, in both of the cohorts, intake of PUFAs was lower than the recommended 5–10 E%. Malnutrition and its risk in the residents was high (> 80%) in both measuring points, which is common in long-term care facilities [5–7]. In order to avoid residents’ weight loss it is a common practice to increase energy content of the served meals by adding fat to the meals during preparation [12]. Traditionally this has meant adding butter or cream to various foods and using whole milk products instead of low fat or fat free products. In the newly published recommendation in Finland targeted especially for older people, fat quality is seen as an important issue, and the use of vegetable oils and soft margarines are encouraged instead of butter and cream [13]. This clearly has not happened in the long-term care settings. Although the data in both cohorts was gathered prior to the publication of the new recommendation, the general recommendation for diet quality has been published in 2014 [15].

Good fat quality may slow some aspects of age-related decline in health. High SFA intakes elevate liver fat and serum cholesterol, whereas increase of MUFA and PUFA seems to be beneficial for modulation of liver fat and lipid metabolism [27–30]. Moreover, high MUFA and PUFA diets may improve insulin sensitivity, [31] reduce type 2 diabetes risk [32] and improve cardiovascular outcomes [33]. Healthy brain is also very much dependent on good cardiovascular health [33]. Fatty acids take part in multiple functions in the body and interact with other dietary components as well as microbiome and thus dietary fat composition may be either pro- or anti-inflammatory [33, 34]. Cardiovascular disease, Alzheimer’s disease and frailty have all been associated with increased chronic inflammation [35–37]. It has been suggested that n-6:n-3 ratio is also important for cardiovascular health [38], although the optimal n-6:n-3 ratio for human health remains under debate [39]. In our study, the n-6:n-3 ratio was reasonable good, but the problem with fat quality had more to do with low PUFA intakes in general. Thus, good fat quality and sufficient intake of n-3 fatty acids are important also for the oldest-old individuals.

Although the residents of the two cohorts were of similar age and did not differ in MNA score or BMI, the residents in the 2017/18 cohort had worse physical and mental health than the residents in the 2007 cohort. This reflects stricter national guidelines for admission to long-term care facilities [40]. In Finland due to public policy institutionalized care for older people have been reduced to the minimum and people are expected to live in their homes as long as possible [38]. Thus, only those who have very severe dementia, mobility disability, or other severe health complications due to multiple chronic diseases, are offered a place in a nursing home or assisted-living facility type of long-term care. This can also been seen in the participants of the 2017/2018 cohort, who had very poor cognition measured with CDR (Table 1) compared to the participants in the 2007 cohort.

However, factors related to nutritional care such as intensive nutritional care, use of oral nutrient supplements, dysphagia, chewing problems, or other nutrition related issues that might be associated with poor nutrition, were not associated with higher SFA intake in this study. Of specific nutritional issues, only dry mouth was inversely associated with SFA intake. This reflects higher food consumption, as those with dry mouth consumed considerably less energy than those who did not. Similarly, residents eating independently and residents reported consuming larger amounts of foods had higher SFA intake than those needing help with eating or those who only reported eating very little. Thus, all the nutritional care related associations were associated with amount of eaten foods.

It is quite interesting, that despite of official recommendations, the fat quality has worsened. This might be because during the past 10 years, specific education on how to identify and treat malnutrition in long-term care has been provided for nurses working in these facilities. This seems to have led to an increment of SFAs to the served foods in order to avoid weight loss of the residents. As sugar intake was one of the strongest predictors of higher SFA intake, it is likely that sugar is also added to the diet in order to make the offered foods more palatable for the residents at risk of weight loss.

The strengths of our study include its large sample of long-term care residents in both 2007 and 2017/18. To best of our knowledge this is the first study that specifically explores detailed fat composition and quality and how it has changed in recent years in the diets of these people. Trained nurses or nutritionists performed all the measurements in both 2007 and 2017/18 cohorts and all the questionnaires and measurements were validated. Moreover, demographic information, and nutritional supplements were retrieved from medical records, which increase the reliability of our results. However, our study also has many limitations. Food diaries may be subject to error. However, since trained nurses filled in the diaries for the residents, subjective under or over reporting is unlikely. More problematically, when only the 1–2 day food diaries are assessed, they may differ from the person’s average food intake over a longer period of time. However, although the individual food intake may vary on a daily basis, our results are relevant at the group level [41]. Moreover, practices that favor using SFA sources such as whole milk products, spreads with high SFA content, butter and cream are not likely to vary considerably from day to day in long-term care facilities. One limitation has to do with the food diary data analyzing tool used in 2007 (Nutrica). Data obtained in the later cohort 2017/18 was more detailed than in 2007, and included also the amounts of trans fatty acids, n-3 and n-6 fatty acids. Therefore, very detailed comparison of fat composition between the two cohorts was not possible. Change in food diary analyzing tools between the two cohorts should not affect the reliability of the dietary intakes, since both programs are based on the same Finnish food database (Fineli) and are validated tools. The residents in the 2017/18 cohort had poorer health, cognition and mobility, which make them more vulnerable to malnutrition compared to the residents of 2007. However, the residents also had many similarities, and did not differ significantly in respect to nutrition between the cohorts. A further limitation in our study is that the mean time of stay in long-term care is only 2 years; therefore it was not possible to follow the same residents over time. However, although changes in dietary fat intake may have been due to numerous confounding factors e.g. dentition, BMI, health status or other dietary factors, in our study higher SFA intakes were only related to the amount of eaten foods.

The 2017/18 sample was randomly selected within voluntary facilities. The latter sample included six long-term care facilities originally included in the 2007 sample. Although, not all the same facilities were compared, all the long-term care facilities are operated by the city of Helsinki and all facilities should follow the same nutritional guidelines. Moreover, they have same resources, same kind of care and residents are alike spending their last years of their lives there. The participation of the facilities in this study was voluntary and the investigators could not influence the participation. Although fewer facilities participated in the cohort on 2017/2018, in fact higher number of residents took part of the study compared to the 2007 study. The results were obtained from long-term care facilities in the Helsinki metropolitan area with residents mostly of Caucasian origin. The data may thus not be applicable to other ethnic groups. Finally, due to observational nature of our study, no causal relationships can be drawn from these results.

## Conclusions

We observed that fat quality has worsened during the last decade among the long-term care residents in spite of the official nutrition recommendations. Nurses and food service personal should be educated about the nutrition recommendations and taught how to increase energy content of foods by using good instead of low quality fats in the diets of long-term residents. This could contribute to better resident health and well-being.

## Data Availability

The dataset analysed during the current study are not publicly available due to privacy regulations of City of Helsinki. Data inquiries should be addressed to professor Kaisu Pitkälä, email: kaisu.pitkala@helsinki.fi

## Abbreviations

BMI: Body mass index
CDR: Clinical dementia rating
EFA: Essential fatty acids
FA: Fatty acid
MNA: Mini Nutritional Assessment
MUFA: Monounsaturated fatty acids
PUFA: Polyunsaturated fatty acids
SFA: Saturated fatty acids
Q: Quartile
